# Whole Organism High Content Screening Identifies Stimulators of Pancreatic Beta-Cell Proliferation

**DOI:** 10.1371/journal.pone.0104112

**Published:** 2014-08-12

**Authors:** Naoki Tsuji, Nikolay Ninov, Mina Delawary, Sahar Osman, Alex S. Roh, Philipp Gut, Didier Y. R. Stainier

**Affiliations:** 1 Department of Biochemistry and Biophysics, Programs in Developmental and Stem Cell Biology, Genetics and Human Genetics, the Diabetes Center, Institute for Regeneration Medicine and Liver Center, University of California San Francisco, San Francisco, California, United States of America; 2 Department of Developmental Genetics, Max Planck Institute for Heart and Lung Research, Bad Nauheim, Germany; 3 DFG Research Center for Regenerative Therapies Dresden, Technische Universität Dresden, Dresden, Germany; 4 Paul Langerhans Institute Dresden, German Center for Diabetes Research, Dresden, Germany; University of Birmingham, United Kingdom

## Abstract

Inducing beta-cell mass expansion in diabetic patients with the aim to restore glucose homeostasis is a promising therapeutic strategy. Although several *in vitro* studies have been carried out to identify modulators of beta-cell mass expansion, restoring endogenous beta-cell mass *in vivo* has yet to be achieved. To identify potential stimulators of beta-cell replication *in vivo*, we established transgenic zebrafish lines that monitor and allow the quantification of cell proliferation by using the fluorescent ubiquitylation-based cell cycle indicator (FUCCI) technology. Using these new reagents, we performed an unbiased chemical screen, and identified 20 small molecules that markedly increased beta-cell proliferation *in vivo*. Importantly, these structurally distinct molecules, which include clinically-approved drugs, modulate three specific signaling pathways: serotonin, retinoic acid and glucocorticoids, showing the high sensitivity and robustness of our screen. Notably, two drug classes, retinoic acid and glucocorticoids, also promoted beta-cell regeneration after beta-cell ablation. Thus, this study establishes a proof of principle for a high-throughput small molecule-screen for beta-cell proliferation *in vivo*, and identified compounds that stimulate beta-cell proliferation and regeneration.

## Introduction

Pancreatic beta-cells secrete insulin which is the only hormone known to directly lower blood glucose concentrations. Type 1 diabetes mellitus is an autoimmune disease characterized by the destruction of beta-cells in the pancreatic islets, leading to insulin deficiency and hyperglycemia. In contrast, type 2 diabetes has a multifactorial origin that commences with insulin resistance and increased serum insulin levels followed by beta-cell destruction, insulin deficiency and hyperglycemia [1], [2]. Restoring functional beta-cell mass is recognized as a promising therapeutic strategy towards normalizing glucose levels in both type 1 and 2 diabetics. Potential strategies for beta-cell mass restoration can generally be categorized as *ex vivo* strategies involving the generation of beta-cells from stem cells, either embryonic stem (ES) cells or induced pluripotent stem (iPS) cells, and their subsequent transplantation, as well as *in vivo* regeneration approaches, including beta-cell mass expansion via reprogramming of other cell types and/or stimulation of proliferation of preexisting beta-cells. It remains unclear which approach will ultimately prove successful, and both approaches may even be synergistic [3].

Although human beta-cell proliferative capacity may decrease with age, replication can be clearly observed in response to metabolic demand, such as in obesity or during pregnancy [4]. Therefore, the identification of means to enhance beta-cell replication is of great interest. However, the regulation of beta-cell proliferation remains poorly understood, partly because of the lack of unbiased approaches to identify the underlying signaling mechanisms. Only recently, a screening platform based on freshly isolated rat islet preparations, which are thought to maintain the metabolic characteristics of primary beta-cells, was used to screen for small molecules that promoted beta-cell replication [5]. This approach identified adenosine kinase inhibitors that promoted the replication of cultured primary beta-cells from mice, rats and pigs. Notably, an independent screen also found an adenosine kinase inhibitor, as well as other positive modulators of adenosine signaling, as potent enhancers of beta-cell regeneration *in vivo* in zebrafish and mice [6]. It is of course important to note that in addition to the artifacts associated with *ex vivo* environments, *in vitro* screens will not identify compounds that stimulate beta-cell proliferation indirectly (e.g., by affecting other cell types in the pancreas or other organs). The zebrafish is thus an ideal model system to carry out large-scale screens, including chemical screens, for beta-cell regeneration [6], beta-cell neogenesis [7] and gluconeogenesis [8]. In this study, we aimed to identify stimulators of beta-cell proliferation *in vivo* via direct quantification of proliferating beta-cells. To achieve this goal, we established an *in vivo* imaging approach utilizing the fluorescent ubiquitylation-based cell cycle indicator (FUCCI) technology [9], [10]. We performed a chemical screen using this approach and identified several small molecules that markedly increased beta-cell proliferation. Importantly, some of these compounds facilitated beta-cell regeneration as well.

## Materials and Methods

### Zebrafish lines

This study was carried out in strict accordance with the NIH guidelines and was approved by the University of California San Francisco Committee on Animal Research. All embryonic dissociations were performed under tricaine anesthesia, and every effort was made to minimize suffering. Zebrafish were raised under standard conditions at 28°C. Phenylthiourea (PTU) was added at 12 hpf to prevent pigmentation. We used the following lines: *Tg(ins:CFP-NTR)^s892^* [11], *Tg(ins:H2BGFP;ins:dsRED)^s960^*,[11], *Tg(ins:kaede)^s949^* [6], *Tg(ins:mCherry-zCdt1(1/190))^s948^, Tg(ins:mAG-zGeminin(1/100))^s949^* [12], *Tg(ins:mAG-zGeminin(1/100))^s947^* (this study; this line was made as previously described [12]).

### Chemical Screening

We bred homozygous *Tg(ins:mAG-zGeminin(1/100))^s947^* with wild-type zebrafish to generate hemizygous *Tg(ins:mAG-zGeminin(1/100))^s947^* animals for chemical screening in order to avoid the variability of fluorescent signal present in a mixture of homozygous and hemizygous transgenics. The eye-marker cassette, *cryaa:RFP* [8] was introduced during the generation of the *Tg(ins:mAG-zGeminin(1/100))* lines in order to facilitate identification of transgenic carriers [12]. Larvae were kept in egg water supplemented with 0.2 mM 1-phenyl-2-thiourea (TCI America) from 1–3 dpf to inhibit pigment formation. Compounds were dissolved in 300 µl of egg water to a final concentration of 1% DMSO and added to the wells of a 96-well plate (Matriplate, 170 um glass bottom, Brooks Life Science Systems). Four larvae were pipetted in 200 µl of egg water and placed in each well, for a final volume of 500 µl, for 1 day of chemical treatment. We screened the following chemical libraries (NIH Clinical Collection 1 and 2 (727 compounds, Evotec), The InhibitorSelect 96-Well Protein Kinase Inhibitor Library II (80 compounds, EMD Millipore), Nuclear receptor ligand library (76 compounds, Enzo Life Sciences)). Two wells, each containing four larvae, were used to evaluate each compound. Initially, we tested the compounds at 10 µM, a routinely used concentration for chemical screens in zebrafish [8]. Compounds that exhibited toxicity at 10 µM, such as those causing pericardial edema or lethality, were retested by gradual reduction of their concentration until a non-toxic dose was identified. At 4 dpf, the larvae were anesthetized with Tricaine, and the number of *Tg(ins*:mAG-zGeminin(1/100))^s947+^ beta-cells was counted using a wide-field Zeiss Z1 inverted microscope with a 20× objective. We counted the number of proliferating beta-cells in each larva by adjusting the focus of the microscope in order to visualize the entire primary islet.

### Immunohistochemistry

Antibody staining was performed as previously described [13], using insulin antibodies (1∶1000, Sigma I8510) and Alexa Fluor conjugated secondary antibodies (1∶500, Invitrogen). Cell nuclei were visualized with TOPRO3 (1∶2000, Invitrogen T3605). Proliferation was assessed by EdU labeling using the Click-iT EdU imaging kit (Invitrogen). Larvae were incubated in 2.5 mM EdU. For confocal analysis, images were captured with a Zeiss LSM5 Pascal confocal microscope. Images were prepared for further examination using ImageJ.

### Blood Glucose Levels

Free glucose was determined by grinding larvae in groups of four and using a glucose assay kit (BioVision) [8]. For blood glucose measurements, adult zebrafish were anesthetized in cold water then decapitated by cutting cleanly through the pectoral girdle with scissors. Whole blood was analyzed immediately with a glucometer test strip (Accu-Check Aviva, Roche Diagnostics) [14].

### Statistical Analysis

Statistical analyses were carried out by two-tailed t-tests and displayed as ±SEM.

## Results

### The FUCCI technology effectively labels proliferating beta-cells

**Figure 1:**
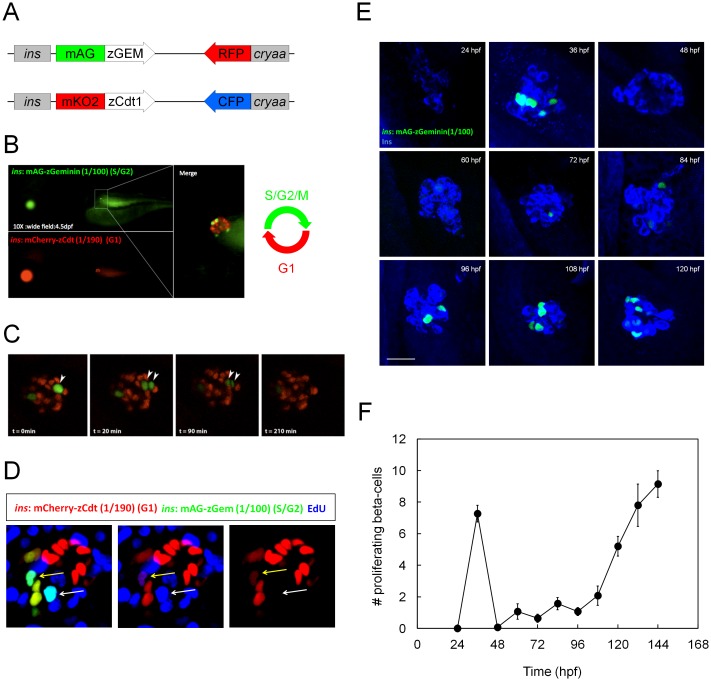
Development and characterization of fluorescent ubiquitylation-based cell cycle indicator (FUCCI) for pancreatic beta-cells in zebrafish. (A) Schematic diagrams of FUCCI constructs for pancreatic beta-cells. The S/G2/M reporter mAG-zGeminin(1/100) and the G1 indicator mKO2-zCdt1(1/190) are expressed under the zebrafish *insulin* promoter. For efficient selection of transgenic animals, an eye-marker cassette, *cryaa:RFP* or *cryaa:CFP*, was introduced into *Tg(ins:mAG-zGeminin(1/100))* and *Tg(ins:mCherry-zCdt1(1/190))*, respectively [8]. (B) *Tg(ins:mCherry-zCdt1(1/190),cryaa:CFP)^s948^;Tg(ins:mAG-zGeminin(1/100),cryaa:RFP)^s947^* larvae were examined at 4.5 dpf using fluorescence microscopy. A close up of the islet is shown in the inset. A majority of the beta-cells are *Tg(ins*:mCherry-zCdt1(1/190))^+^ indicating that they are in the G1 phase of the cell cycle. Only four beta-cells are *Tg(ins*:mAG-zGeminin(1/100))*^s947^* ^+^ indicating that they are in the S/G2/M phase of the cell cycle. Note that the animals are expressing the eye-marker, e.g., *cryaa*:CFP fluorescence can be observed through the GFP filter. (C) Time-lapse imaging of *Tg(ins:mCherry-zCdt1(1/190))^s948^;Tg(ins:mAG-zGeminin(1/100))^s947^* larvae at 4 dpf. Arrowheads point to dividing *Tg(ins*:mAG-zGeminin(1/100))*^s947^* ^+^ beta-cells. (D) *Tg(ins:mCherry-zCdt1(1/190))^s948^;Tg(ins:mAG-zGeminin(1/100))^s947^* larvae were incubated with EdU from 3 to 4 dpf. The white arrow points to a *Tg(ins*:mAG-zGeminin(1/100))*^s947^* ^+^ single-positive beta-cell. This cell exhibits high levels of EdU incorporation. The yellow arrow points to a *Tg(ins*:mCherry-zCdt1(1/190))*^s948^;Tg(ins*:mAG-zGeminin(1/100))*^s947^* double positive beta-cell which exhibits low levels of EdU incorporation indicating that this cell entered S phase at the end of the EdU labeling period. (E) Confocal stacks of *Tg(ins*:mAG-zGeminin(1/100))*^s947^* ^+^ (green) beta-cells stained for Insulin (blue). The animals were fixed at 12 h intervals until 5 dpf. (Scale bar = 20 µm.). (F) The graph shows a quantification of the number of *Tg(ins*:mAG-zGeminin(1/100))*^s947^* ^+^ beta-cells. Error bars represent SEM; n = 13–15 larvae for each time point. B is a lateral view, anterior to the left and dorsal to the top. C–E show lateral views, anterior to the top and dorsal to the left.

In order to be able to quantitate pancreatic beta-cell proliferation *in vivo*, we utilized recently generated zebrafish transgenic lines expressing mCherry-zCdt1(1/190) (a G1 marker) and mAG-zGeminin(1/100) (an S/G2/M marker) under the control of the *insulin* regulatory elements [12] (Fig. 1A). Using live imaging and 5-ethynyl-2′-deoxyuridine (EdU) incorporation analyses, we found that the *Tg(ins:mAG-zGeminin(1/100))^s946^* and *Tg(ins:mCherry-zCdt1(1/190))^s948^* lines mark the proliferating and quiescent beta-cells, respectively, and that *Tg(ins*:mAG-zGeminin(1/100))*^s946^* expression disappears several minutes after mitosis [12]. In order to facilitate the counting of proliferating beta-cells using a fluorescence microscope, we generated *Tg(ins:mAG-zGeminin(1/100))^s947^*, a new line that exhibits a much brighter fluorescent signal in beta-cells compared to the previous line (Fig. 1B). This effect is very likely due to a higher basal level of expression of the *insulin* promoter as a result of a more favorable genomic integration site and/or a higher number of transgenic concatemers. Importantly, using live imaging, we found that the dividing beta-cells maintained visible levels of *Tg(ins*:mAG-zGeminin(1/100))*^s947^* expression in their daughter cells for up to several hours after mitosis (352±175 minutes, n = 3 dividing cells in 2 movies) (Fig. 1C). We reasoned that for the purposes of a chemical screen, this line provided a more sensitive readout of beta-cell proliferation. It allows one to score not only the beta-cells in the S/G2/M phase of the cell cycle but also those that divided several hours before the end of the compound-treatment period. In addition and consistent with our previous observations [12], *Tg(ins*:mAG-zGeminin(1/100))*^s947^* single positive beta-cells readily incorporated EdU whereas the *Tg(ins*:mAG-zGeminin(1/100));*Tg(ins*:mCherry-zCdt1(1/190)) double-positive beta-cells were either EdU^−^ or weakly EdU^+^, indicating that they were in an early stage of S-phase (Fig. 1D).

### High levels of beta-cell proliferation occur during two stages of early zebrafish development

The temporal regulation of beta-cell proliferation in zebrafish has not been studied in detail during the embryonic and early larval stages. To address this issue, we first analyzed *Tg(ins:mAG-zGeminin(1/100))^s947^* animals during narrow developmental intervals (12 h) from 24 to 144 hpf in live and fixed preparations (Fig. 1E). Interestingly, we found that during early development, there are two peaks of proliferation in pancreatic beta-cells, one around 36 hpf and the other around 120 hpf (Fig. 1F). As a temporal reference point, the heart starts beating at around 24 hpf [15]. These two peaks coincide with the two periods when the animals exhibit an increase in glucose levels [8], [16], suggesting that high glucose levels stimulate a compensatory increase in beta-cell number. Whether glucose exerts a direct effect on beta-cell proliferation or whether the increase in proliferation is the result of transient insulin resistance during these two periods, as shown in mouse models [5], [16], remains to be determined. Importantly, these analyses also revealed that from 72 to 96 hpf, a majority of beta-cells enter a period of quiescence.

### *In vivo* high content screening identifies stimulators of pancreatic beta-cell proliferation

**Figure 2:**
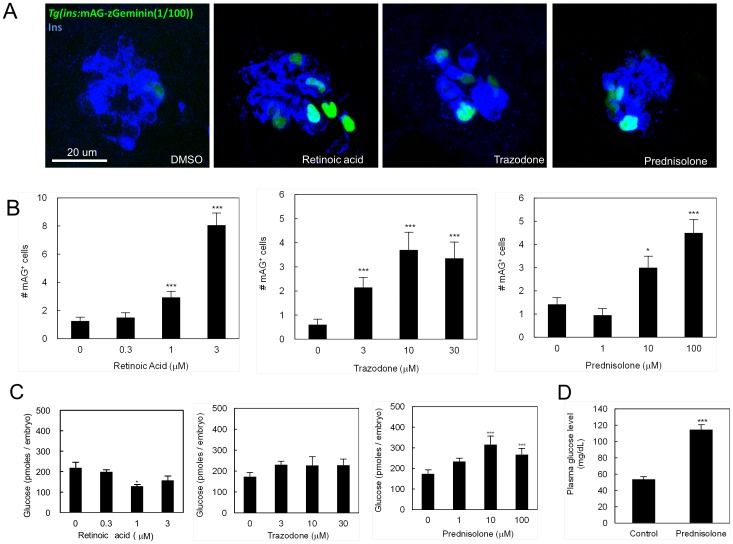
Retinoic acid and Trazodone promote beta-cell proliferation without inducing hyperglycemia. (A) Confocal images of *Tg(ins*:mAG-zGeminin(1/100))*^s947^* ^+^ beta-cells (green) stained for Insulin (blue). The animals were treated with 1 µM retinoic acid, 10 µM trazodone or 10 µM prednisolone in 1% DMSO (scale bar = 20 µm). (B) Dose–response curves for *Tg(ins*:mAG-zGeminin(1/100))*^s947^* ^+^ beta-cells showing the relationship between the concentration of the compounds and the number of proliferating beta-cells in larvae treated with retinoic acid, trazodone, or prednisolone in 1% DMSO from 3 to 4 dpf. Error bars represent SEM. *P<0.05, and ***P<0.005 compared to vehicle-treated controls; n = 16–20 larvae for each group. (C) Absolute glucose values in zebrafish larvae treated with retinoic acid, trazodone, or prednisolone in 1% DMSO. Error bars represent SEM. *P<0.05, and ***P<0.005 compared to vehicle-treated controls; n = 16–20 larvae for each group. (D) Blood glucose concentration in adult zebrafish treated with 30 µM prednisolone for 24 h. Error bars represent SEM. ***P<0.005 compared to vehicle-treated controls; n = 10 fish for each group.

With this *in vivo* platform based on the FUCCI reporter, we designed a screening protocol of low molecular weight compounds to find ones that enhance beta-cell proliferation. A typical time series of *Tg(ins:mAG-zGeminin(1/100))* ^s947^ larvae is shown in Figs. 1E and F. For chemical screening, we chose the developmental period during which most beta-cells are in a quiescent state, in order to identify compounds that stimulate their proliferation. We arrayed hemizygous *Tg(ins:mAG-zGeminin(1/100))^s947^* 72 hpf larvae in 96-well plates, and exposed them to chemicals from selected libraries of bioactive compounds as shown in Fig. S1. After 24 hours of chemical exposure, the number of proliferating beta-cells in the primary islet was counted. A typical example of *Tg(ins*:mAG-zGeminin(1/100))*^s947^* ^+^ beta-cell counting using an inverted fluorescence microscope is shown in Fig. S1. At this developmental stage, the basal *in vivo* beta-cell proliferation count showed moderate inter-experiment variability (0.53±0.04 cells per larva, n = 788). A compound that showed a statistically significant increase in the number of *Tg(ins*:mAG-zGeminin(1/100))*^s947^* ^+^ beta-cells compared to DMSO-treated control was regarded as a “hit”. We screened 883 compounds for their ability to induce beta-cell proliferation. The primary screen and the subsequent secondary validation identified 20 hit compounds (2.27% hit rate) (Table 1). Notably, all hits could be grouped into three common pharmacological classes, namely retinoid receptor agonists, enhancers of serotonin signaling, and glucocorticoids (Table 1). We further tested representative compounds for each pathway; retinoic acid, trazodone (serotonin uptake inhibitor), and prednisolone acetate (glucocorticoid) (Fig. 2A). All of these compounds induced replication of beta-cells in a dose-dependent manner with the following effective concentrations to achieve a 2-fold increase in proliferation (EC_TFP_): ≈0.67, 1.11, and 10.3 µM, respectively (Fig. 2B). Moreover, these compounds increased the number of beta-cells in S-phase, as assessed by EdU incorporation (Fig. S2).

**Table 1:** List of compounds that increase the number of proliferating beta-cells in *Tg(ins:mAG-zGeminin(1/100))^s947^* larvae.

Classification	Chemical Name	# mAG^+^ cells	Pharmacological Action	Dose (µM)	Library
Control	DMSO	0.5±0.06			
Retinoic acid	Retinoic acid, all trans	5.0±0.7	RAR agonist	10	NRL
	4-Hydroxyretinoic acid	3.7±0.9	Retinoid metabolite	10	NRL
	9-cis Retinoic acid	2.5±0.5	RXR agonist	10	NRL
	Isotretinoin (13-cis-Retinoic Acid)	2.5±0.5	RAR agonist	10	NIH II
Serotonin	Trazodone	1.3±0.3	Serotonin uptake inhibitor	10	NIH I
	Lofepramine	1.0±0.3	Serotonin and noradrenalin re-uptake inhibitor	10	NIH I
	Phenelzine	1.1±0.3	Monoamine oxidase inhibitor	10	NIH II
Glucocorticoids	Fluticasone	2.7±0.6	Glucocorticoid receptor ligands	10	NIH I
	Hydrocortisone acetate	2.8±0.7		10	NIH II
	Prednisolone acetate	2.9±0.7		10	NIH II
	Clobetasol propionate	2.7±0.5		10	NIH II
	Triamcinolone acetonide	2.9±0.5		10	NIH II
	Westcort	2.6±0.4		10	NIH II
	Budesonide	3.2±0.4		10	NIH II
	Methylprednisolone acetate	2.0±0.4		10	NIH II
	Fluocinolone acetonide 21-acetate	2.3±0.4		10	NIH II
	Dexamethasone	1.4±0.3		10	NIH II
	Cortisone acetate	1.2±0.3		10	NIH II
	Amcinonide	2.1±0.4		10	NIH II
	Fluocinolone acetonide	1.7±0.3		10	NIH II

The hits are organized into three categories based on their pharmacological action. All hits showed a statistically significant increase in beta-cell proliferation compared to DMSO controls (n = 12 to 20 animals for each compound); p<0.05.

Next, we tested the effect of the hits on glucose levels because high glucose levels are known to increase beta-cell mass *in vitro* [17] and *in vivo* [18]. By measuring free glucose, i.e., glucose that has not been phosphorylated intracellularly by hexokinases, we estimated glycemia [6], [8]. Using this assay, we found that free glucose levels were not elevated by the treatment with retinoic acid, or trazodone, but prednisolone did significantly increase glucose levels (Fig. 2C). To further analyze the effect of prednisolone on glucose levels, we treated adult fish. We found that plasma glucose levels were two folds higher after a 24 hour exposure to prednisolone compared to controls (Fig. 2D), suggesting that the induction of beta-cell proliferation by prednisolone was caused by hyperglycemia. Thus, the proliferation effect of glucocorticoids is likely indirect, i.e., via increased glucose levels, in agreement with previous studies [19].

### The hit compounds promote beta-cell proliferation under feeding metabolism

**Figure 3:**
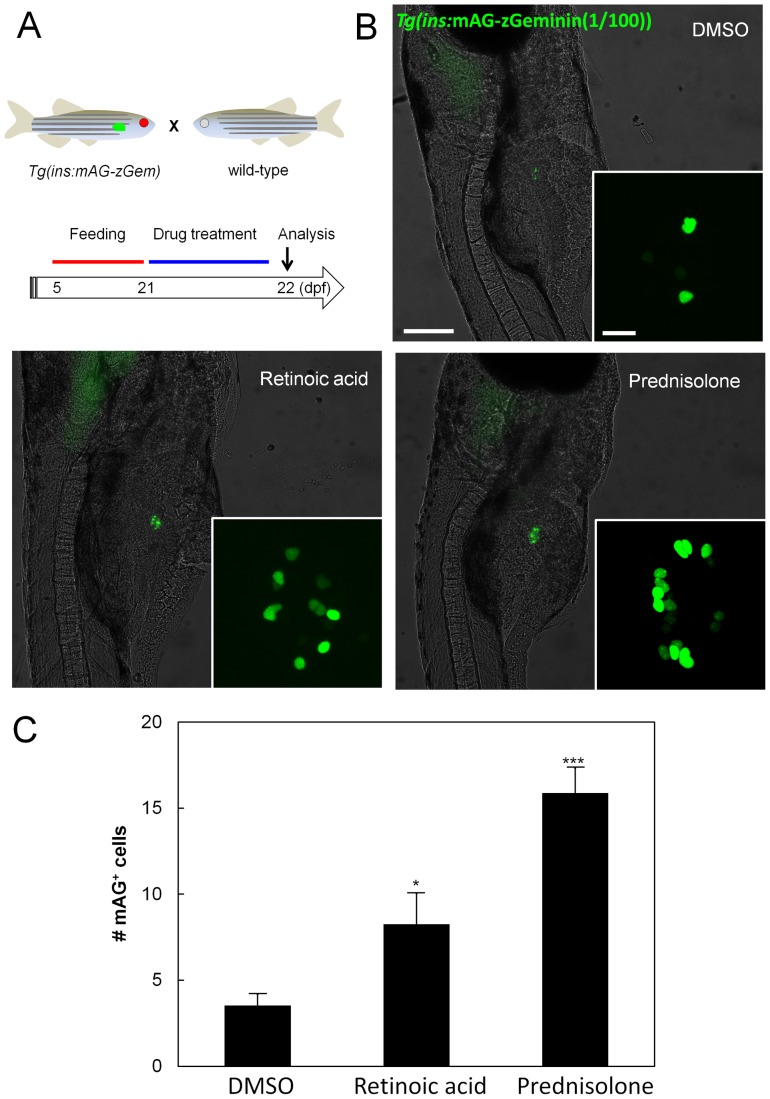
RA and Prednisolone effectively increase beta-cell proliferation under feeding metabolism. (A) Schematic diagram for assessment of beta-cell proliferation. At 21 dpf, after feeding from 5–21 dpf with Kyowa N-250 (Kyowa), *Tg(ins:mAG-zGeminin(1/100))^s947^* larvae were treated with 1 µM retinoic acid or 10 µM prednisolone in 1% DMSO. The number of *Tg(ins*:H2BGFP)*^+^* cells was counted at 22 dpf. (B) *Tg(ins*:mAG-zGeminin(1/100))*^s947^* ^+^ beta-cells were examined at 22 dpf using an epifluorescence microscope. Scale bar = 200 µm. A close up of the islet examined using a confocal microscope is shown in the inset. Scale bar = 10 µm. (C) Quantification of proliferating beta-cells per larva at 22 dpf. Error bars represent SEM. *P<0.05, and ***P<0.005 compared to DMSO-treated controls; n = 13–16 animals for each group.

The chemical screening was carried out with animals during the early larval stage. During this stage, the animals rely on yolk consumption for energy production and enter a fasting state by 4–5 dpf [8]. To test the capability of the hit compounds to promote beta-cell proliferation under a more active metabolism, we evaluated the effect of representative hits of two of the more potent classes (prednisolone and retinoic acid) during the late larval stages (Fig. 3A), when the animals are feeding externally. The number of *Tg(ins*:mAG-zGeminin(1/100))*^s947^* ^+^ beta-cells was significantly higher in animals treated with the compounds compared to those treated with DMSO (Figs. 3B and 3C), indicating that they can stimulate proliferation at later developmental stages and under feeding metabolism.

### The hit compounds promote beta-cell regeneration

**Figure 4:**
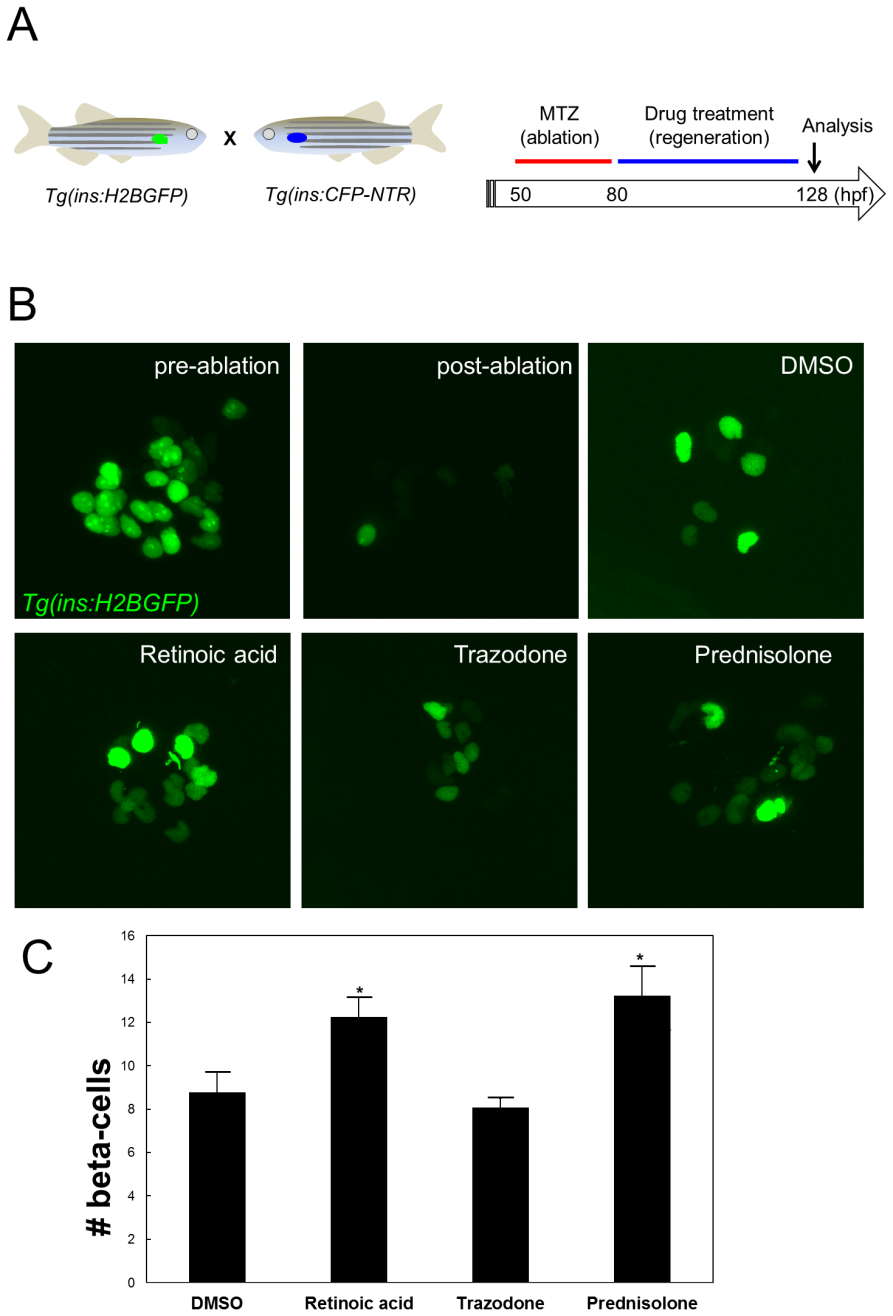
RA and Prednisolone but not Trazodone, enhance beta-cell regeneration. (A) Schematic diagram for assessment of beta-cell regeneration. To examine beta-cell regeneration, we made use of the NTR/MTZ beta-cell ablation model. At 80 hpf, after ablating the beta-cells with MTZ from 50–80 hpf, *Tg(ins:H2BGFP);Tg(ins:CFP-NTR)* larvae were treated with the compounds for 48 h. The numbers of *Tg(ins*:H2BGFP)^+^ cells were counted at 128 hpf. (B) Confocal images of *Tg(ins*:H2BGFP)^+^ beta-cells in larvae treated with 1 µM retinoic acid, 10 µM trazodone, or 10 µM prednisolone in 1% DMSO at 128 hpf. Each image is a lateral view, anterior to the bottom and dorsal to the right. (C) Quantification of beta-cell regeneration per larva at 128 hpf, following treatment with hit compounds from 80–128 hpf. Error bars represent SEM. *P<0.05 compared to DMSO treated controls; n = 13–16 larvae for each group.

We also investigated whether the hit compounds could compensate for beta-cell loss in a diabetic model [11], [20]. In this model, beta-cells are ablated via cell-specific transgenic expression of nitroreductase (NTR), an enzyme that converts the chemical metronidazole (MTZ) to a cell lethal product. We exposed *Tg(ins:CFP-NTR);Tg(ins:H2BGFP)* larvae to MTZ from 50 to 80 hpf, followed by a wash out of the drug (Fig. 4A). Typically, MTZ-treated larvae exhibit only rare beta-cells after 24 hours in MTZ (Fig. 4B). After allowing beta-cells to regenerate for 2 days in the presence of each hit compound, we analyzed beta-cell regeneration by counting the number of H2BGFP^+^ beta-cells. In DMSO-treated larvae, 8.7±1.0 beta-cells were present 2 days after ablation (Figs. 4B and 4C). Strikingly, retinoic acid and prednisolone significantly increased the number of beta-cells to 12.3 and 13.2, respectively (Figs. 4B and 4C), indicating that the hit compounds can also promote beta-cell regeneration. Interestingly, trazodone, which only mildly increased beta-cell proliferation in the absence of beta-cell ablation, failed to promote beta-cell regeneration (Figs. 4B and 4C). We conclude that the compounds that induce a potent increase in beta-cell proliferation under physiological conditions can also stimulate beta-cell regeneration consistent with the notion that enhancing beta-cell proliferation can increase regeneration as was shown for the nonselective adenosine agonist NECA [6].

## Discussion

In this study we identified 20 compounds that increased beta-cell proliferation in zebrafish. Interestingly, despite using an unbiased screening approach of over 800 compounds, we found that several compounds converge on common pharmacological classes, namely retinoid receptor agonists, enhancers of serotonin signaling, and glucocorticoids.

Among the retinoid receptor agonists, retinoic acid is well known to play a crucial role shortly after gastrulation in endoderm patterning and pancreatic development, a role which is conserved from zebrafish to humans [21]–[23]. This role in pancreatic induction has also been examined during mouse and human ES cell differentiation *in vitro*, and has been shown to be relevant for the induction of the pancreatic endoderm marker, PDX1 [24]–[26]. Although little is known about the proliferation effect of retinoic acid, recent studies have shown that retinoic acid can increase the proliferation of cultured human pancreatic progenitor cells [27] and the number of pancreatic beta-cells in zebrafish [28].

Serotonin has been widely studied as a brain neurotransmitter for its effects on appetite and mood, especially depression. A recent analysis of pregnant and non-pregnant mice revealed that serotonin acts downstream of lactogen signaling to stimulate beta-cell proliferation during pregnancy [29]. Moreover, serotonin is highly expressed in mammalian beta-cells [30]. Identification of several serotonin signaling enhancers from our screen, including trazodone (a serotonin uptake inhibitor) is consistent with this role. Interestingly, trazodone did not promote beta-cell regeneration in our beta-cell ablation model. This lack of effect on regeneration might be due to a greatly reduced serotonin pool after the ablation of the serotonin-expressing beta-cells or to its lower capacity to induce beta-cell proliferation compared to retinoic acid and prednisolone.

The consistency between our results and previous data (e.g., beta-cell proliferation induced by serotonin and glucocorticoid) establishes the validity of our *in vivo* screen based on the FUCCI technology to identify relevant pathways in beta-cell proliferation. In the past decade, the zebrafish has emerged as a viable model organism for small-molecule discovery. Using zebrafish, it is now possible to assess the specificity, efficacy, and toxicity of small molecules in the context of live animals [31]. By using zebrafish for chemical screening, we identified candidates that induced beta-cell proliferation. The next step is to implement a system for automated image analysis in order to achieve a high throughput screening of beta-cell proliferation *in vivo*. This strategy could make a significant contribution for a primary drug screening for inducers of beta-cell proliferation, identifying not only direct inducers but also indirect ones, i.e., compounds that would affect beta-cell proliferation through cell-cell interaction and/or inter-organ crosstalk. Since current *in vitro* screening approaches cannot identify such indirect inducers of beta-cell proliferation [32]–[34], our platform can be expected to find novel drug candidates. It must be noted of course, that tests with human cells and tissues will be necessary to further evaluate the ability of the hits from such screens in zebrafish to induce the formation of functional beta-cells.

## Supporting Information

Figure S1 **Schematic outline of the screening protocol used to identify compounds that promote beta-cell proliferation.** The images show typical examples from the screen. *Tg(ins:mAG-zGeminin(1/100))^s947^* larvae were arrayed in 96-well plates and exposed to 10 µM of a compound in 1% DMSO from 3 to 4 dpf (i.e., when most beta-cells are in a resting phase (Fig. 1F)). Larvae were incubated in 1% DMSO as a negative control. *Tg(ins*:mAG-zGeminin(1/100))*^s947^* ^+^ beta-cells in 4 dpf anesthetized larvae were counted by eye under an inverted fluorescence microscope. Beta-cell proliferation can be easily quantified because mAG-zGeminin(1/100) labels the nuclei of proliferating beta-cells with bright fluorescence. Fluorescent image at the bottom panel is a lateral view, anterior to the left and dorsal to the top (Scale bar = 100 µm).
(TIF)

Figure S2 **The hit compounds increase the number of beta-cells undergoing S-phase.** (A) *Tg(ins:Kaede)* larvae were treated from 3 to 5 dpf with 1% DMSO, 1 µM retinoic acid, 10 µM trazodone, or 10 µM prednisolone in the presence of 2.5 mM EdU. The numbers of *Tg(ins*:Kaede)+(green) and EdU+ (red) beta-cells were increased in the animals treated with the hit compounds as compared to DMSO-controls. (B) Quantification of the number of *Tg(ins*:Kaede)+and EdU+ beta-cells. Retinoic acid (n = 18 animals) and prednisolone (n = 16 animals) significantly increased the number of EdU+ beta-cells compared to DMSO controls (n = 15 animals). Trazodone (n = 17 animals) only mildly increased the number of EdU+ beta-cell compared to DMSO controls consistent with its less potent effect on beta-cell proliferation (see Table 1); this effect was not statistically significant (N.S.) (p = 0.46). *p<0.05 and **p<0.01. Error bars represent SEM.
(TIF)
