# Supplementary figures and images for: Whole Organism High Content Screening Identifies Stimulators of Pancreatic Beta-Cell Proliferation
Source: PLoS One. 2014 Aug 12;9(8):e104112. doi: 10.1371/journal.pone.0104112 (PMC4130527; doi:10.1371/journal.pone.0104112)

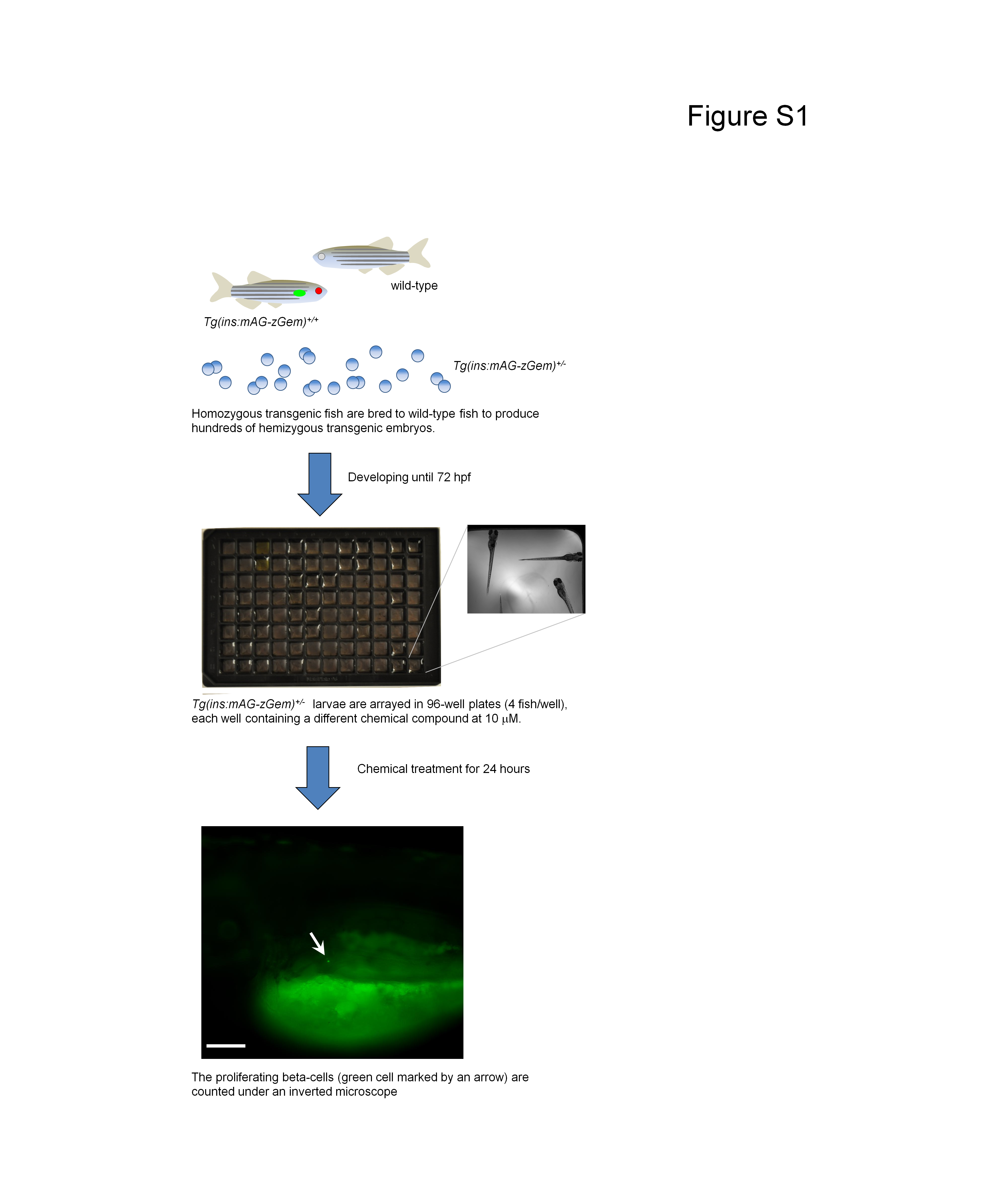

Supplement: Figure S1 — Schematic outline of the screening protocol used to identify compounds that promote beta-cell proliferation. The images show typical examples from the screen. Tg(ins:mAG-zGeminin(1/100))s947 larvae were arrayed in 96-well plates and exposed to 10 µM of a compound in 1% DMSO from 3 to 4 dpf (i.e., when most beta-cells are in a resting phase (Fig. 1F)). Larvae were incubated in 1% DMSO as a negative control. Tg(ins:mAG-zGeminin(1/100))s947 + beta-cells in 4 dpf anesthetized larvae were counted by eye under an inverted fluorescence microscope. Beta-cell proliferation can be easily quantified because mAG-zGeminin(1/100) labels the nuclei of proliferating beta-cells with bright fluorescence. Fluorescent image at the bottom panel is a lateral view, anterior to the left and dorsal to the top (Scale bar = 100 µm). (TIF) [file pone.0104112.s001.tif]

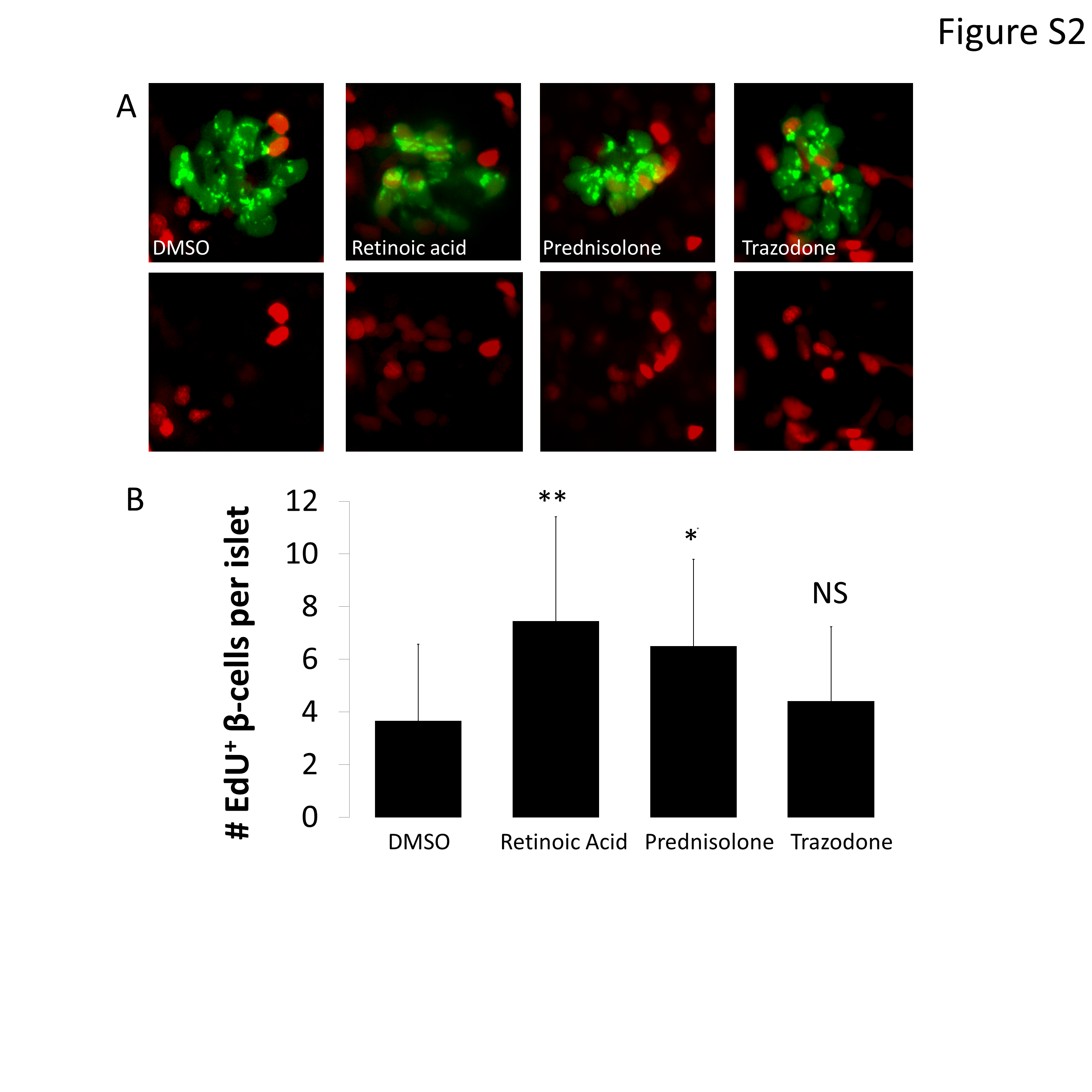

Supplement: Figure S2 — The hit compounds increase the number of beta-cells undergoing S-phase. (A) Tg(ins:Kaede) larvae were treated from 3 to 5 dpf with 1% DMSO, 1 µM retinoic acid, 10 µM trazodone, or 10 µM prednisolone in the presence of 2.5 mM EdU. The numbers of Tg(ins:Kaede)+(green) and EdU+ (red) beta-cells were increased in the animals treated with the hit compounds as compared to DMSO-controls. (B) Quantification of the number of Tg(ins:Kaede)+and EdU+ beta-cells. Retinoic acid (n = 18 animals) and prednisolone (n = 16 animals) significantly increased the number of EdU+ beta-cells compared to DMSO controls (n = 15 animals). Trazodone (n = 17 animals) only mildly increased the number of EdU+ beta-cell compared to DMSO controls consistent with its less potent effect on beta-cell proliferation (see Table 1); this effect was not statistically significant (N.S.) (p = 0.46). *p<0.05 and **p<0.01. Error bars represent SEM. (TIF) [file pone.0104112.s002.tif]
